# Identification of Candidate Biomarkers for Salt Sensitivity of Blood Pressure by Integrated Bioinformatics Analysis

**DOI:** 10.3389/fgene.2020.00988

**Published:** 2020-09-03

**Authors:** Chen Chen, Guan-Zhi Liu, Yue-Yuan Liao, Chao Chu, Wen-Ling Zheng, Yang Wang, Jia-Wen Hu, Qiong Ma, Ke-Ke Wang, Yu Yan, Yue Yuan, Jian-Jun Mu

**Affiliations:** ^1^Department of Cardiology, The First Affiliated Hospital of Medical School, Xi’an Jiaotong University, Xi’an, China; ^2^Key Laboratory of Molecular Cardiology of Shaanxi Province, Xi’an, China; ^3^Key Laboratory of Environment and Genes Related to Diseases, Xi’an Jiaotong University, Ministry of Education, Xi’an, China; ^4^Bone and Joint Surgery Center, The Second Affiliated Hospital of Xi’an Jiaotong University, Xi’an, China

**Keywords:** salt sensitivity of blood pressure, biomarker, circRNA, hub gene, weighted gene coexpression network analysis, bioinformatics

## Abstract

In the current study, we aimed to identify potential biomarkers for salt sensitivity of blood pressure (SSBP), which may provide a novel insight into the pathogenic mechanisms of salt-sensitive hypertension. Firstly, we conducted weighted gene coexpression network analysis (WGCNA) and selected a gene module and 60 hub genes significantly correlated to SSBP. Then, GO function and KEGG signaling pathway enrichment analysis and protein–protein interaction (PPI) network analysis were performed. Furthermore, we identified a five-gene signature with high connectivity degree in the PPI network and high AUC of ROC curves, which may have high diagnosis value for SSBP. Moreover, through combining two gene screening methods, we identified 23 differentially expressed circRNAs and selected the top 5% circRNAs (1 circRNA) with the highest connectivity degree in the coexpression network as hub circRNA highly associated with SSBP. Finally, we carried out RT-qPCR to validate the expression of five hub genes, and our results showed that the expression of HECTD1 (*P* = 0.017), SRSF5 (*P* = 0.003), SRSF1 (*P* = 0.006), HERC2 (*P* = 0.004), and TNPO1 (*P* = 0.002) was significantly upregulated in the renal tissue in salt-sensitive rats compared to salt-resistant rats, indicating that these five hub genes can serve as potential biomarkers for SSBP.

## Introduction

Salt sensitivity of blood pressure (SSBP) is a pathophysiological trait in some members of the population in whom blood pressure will exhibit a change parallel to the load of salt intake (Elijovich et al., 2016). An important issue is that it represents an abnormal phenotype that is commonly manifested in various species, and it has been demonstrated that SSBP is a risk factor for cardiovascular morbidity and mortality, independent of blood pressure (Morimoto et al., 1997; Weinberger et al., 2001). Previous research has suggested that the disruption of salt balance in salt-sensitive individuals, which should be maintained by natriuretic and antinatriuretic systems, is involved in SSBP and salt-sensitive hypertension (Tiu et al., 2017). In addition, multiple abnormalities in the renin–angiotensin–aldosterone system, endothelial system, NO, oxidative stress, sympathetic nerve system, and insulin resistance are considered to play important roles in the occurrence of SSBP and salt-sensitive hypertension (Cappuccio et al., 1985; Hoffman et al., 1994; Yatabe et al., 2010; Laffer and Elijovich, 2013). Gene polymorphism is another potential mechanism, with proofs from different racial and ethnic groups, such as the Genetic Epidemiology Network of Salt Sensitivity (GenSalt) study (GenSalt Collaborative Research Group, 2007). The heritability of SSBP has been estimated at approximately 74% in African Americans and 50% in Chinese (Svetkey et al., 1996; Gu et al., 2007). Nevertheless, the ultimate, precise pathogenesis mechanisms of SSBP remain elusive.

It has been reported that less than 2% of the total genome encodes protein-coding genes and that non-coding RNAs (ncRNAs) represent the majority of the human transcriptome (Fiedler et al., 2018; Leimena and Qiu, 2018). Circular RNAs (circRNAs) are a group of non-coding RNAs generated by a form of alternative splicing (AS), called back-splicing (Kristensen et al., 2019; Xiao et al., 2020). CircRNAs are suggested to play crucial roles in a large number of diseases and physiological processes, such as cardiac hypertrophy and hypertension (Dong et al., 2018; Leimena and Qiu, 2018). One of the main functional mechanisms of circRNA is to regulate gene expression due to its miRNA sponge ability, serving as competitive endogenous RNA (ceRNA) (Qu et al., 2015; Hsiao et al., 2017). However, little attention has been focused on the circRNA and mRNA biomarkers. Hence, there is an urgent need to identify effective biomarkers for SSBP.

Weighted gene coexpression network analysis (WGCNA) is an advanced bioinformatics method for the identification of highly correlated gene modules and hub genes based on a network-focused algorithm (Zhang and Horvath, 2005). Compared with the traditional differentially expressed gene (DEG) screening algorithm, which only takes the significance of a single gene into account, WGCNA constructs a scale-free coexpression network and then clusters genes into several modules according to their relevance. Finally, WGCNA can identify the phenotype-associated modules and hub genes.

In the current study, we combined WGCNA and DEG approaches as well as coexpression network analysis to identify hub circRNAs and hub genes highly associated with SSBP. Besides, we carried out RT-qPCR to validate the expression of five upregulated hub genes that may serve as potential biomarkers for SSBP. These analyses provided a comprehensive understanding about the circRNA and gene expression profile of SSBP at the transcriptome level.

## Results

### Data Preprocessing and Construction of the Weighted Coexpression Networks

**FIGURE 1:**
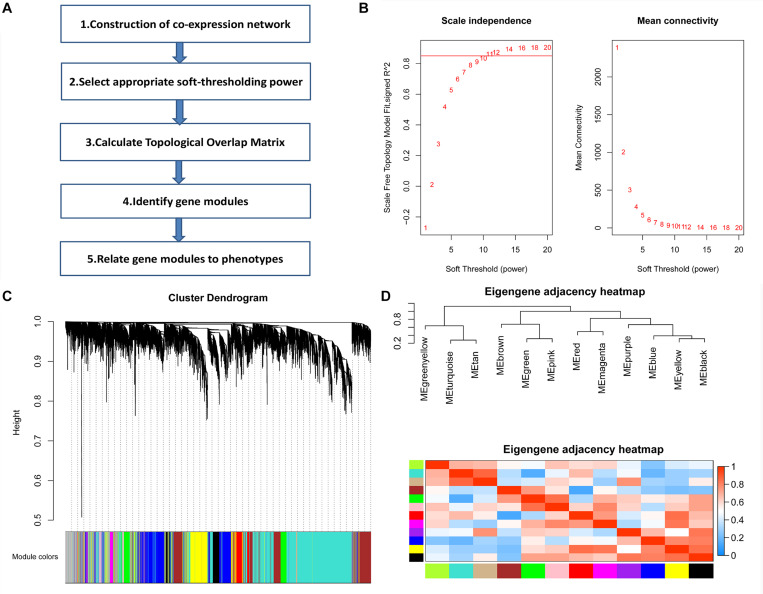
Weighted gene coexpression network analysis (WGCNA). **(A)** The schematic of WGCNA. **(B)** Determination of the soft threshold. Analysis of the scale-free topology fitting index *R*^2^ (left) and mean connectivity (right) for various soft threshold powers. The red line in the left panel indicates *R*^2^ = 0.85. **(C)** Clustering diagram showing 12 modules represented by different colors. **(D)** Clustering tree of module eigengenes and the heatmap of the correlation between any two module eigengenes.

The GSE12424 dataset in the Gene Expression Omnibus (GEO) database includes 10 SS/jr rat kidney gene expression profiles and 8 congenic substrain S.R rat (D3Mco36-D3Mco46) kidney gene expression profiles as well as 8 congenic substrain S.R rat (D3Mco36-D3Got166) kidney gene expression profiles of 10,963 genes. We selected the top 75% of genes with the highest mean absolute deviation (MAD) for further analysis. Then, we calculated the soft-thresholding power β = 11 to establish a scale-free (scale free *R*^2^ > 0.85) coexpression network. Then, through average linkage hierarchical clustering method, these genes were clustered into 12 modules. Every module was labeled with different colors, and its expression level was represented by the corresponding module eigengene (ME) (Figure 1).

### Identification of Significant Modules and Hub Genes

**FIGURE 2:**
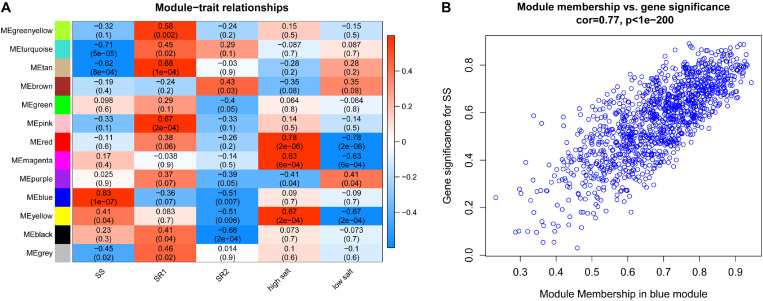
**(A)** Heatmap of the module–trait relationship: red for positive correlation and blue for negative correlation. **(B)** Scatter diagrams of genes in the blue module. The *x*-axis represents gene significance, and the *y*-axis represents module membership.

In this study, we performed correlation analysis between these 12 modules and several clinical phenotypes, including SSBP, salt-resistant type 1 (S.R rat with QTL2 mentioned above), salt-resistant type 2 (S.R rat with both QTL1 and QTL2), high-salt diet, and low-salt diet. Our results indicated that the genes in the blue module were highly associated with SSBP (Figure 2A). The top 5% of the genes (60 genes) with the highest connectivity degree in the blue module were selected as hub genes. Hence, we identified the blue module as a key module and 60 genes as hub genes associated with SSBP. Each gene’s gene significance (GS) and module membership (MM) were calculated and plotted in a scatter diagram (Figure 2B).

### Functional Enrichment Analysis

**FIGURE 3:**
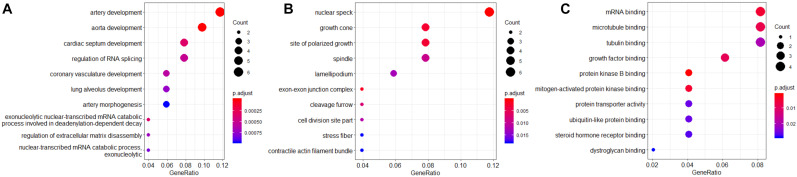
Gene Ontology functional enrichment analysis for biological process (BP) **(A)**, molecular function (MF) **(B)**, and cellular component (CC) **(C)**. The color gradient represents the *P*-value, and the spot size represents the gene number.

Gene Ontology (GO) functional enrichment analysis for these 60 hub genes was performed. The results indicated that these hub genes were significantly enriched in biological process (BP), such as artery development, cardiac development, and regulation of RNA splicing (Figure 3A). For molecular functions (MFs), these hub genes were mainly involved in mRNA binding, microtubule binding, and tubulin binding (Figure 3B). In addition, these hub genes were most significantly enriched in the cellular component (CC) of nuclear speck (Figure 3C).

### Protein–Protein Interaction Network Construction

**FIGURE 4:**
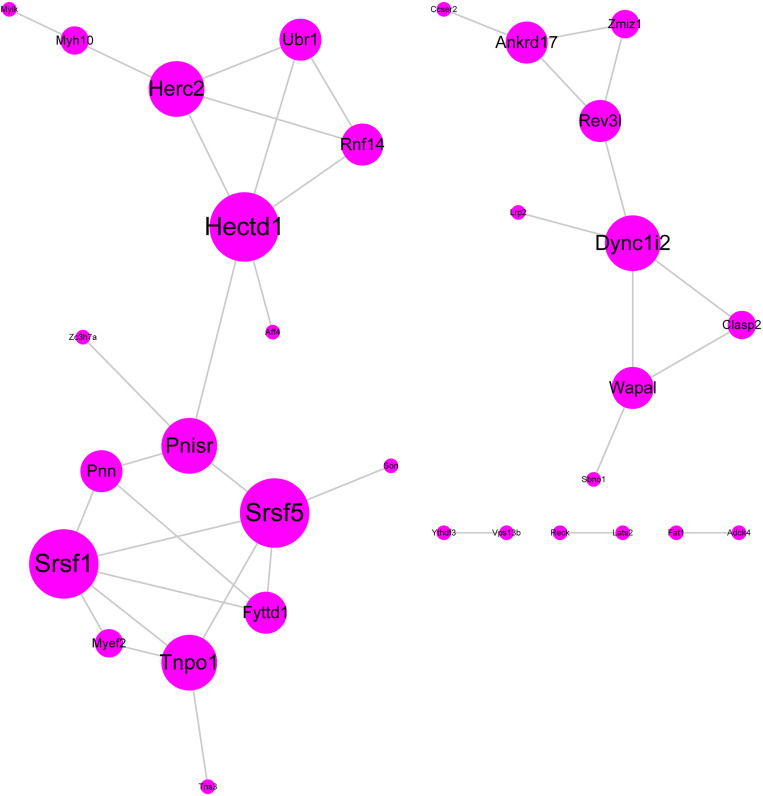
Protein–protein interaction network. The color gradient and spot size represent the connectivity degree.

We performed protein–protein interaction (PPI) analysis by the STRING database, and a PPI network for these 60 hub genes was established (Figure 4). In this PPI network, we found some hub genes with high connectivity degree: HECTD1 (HECT domain E3 ubiquitin protein ligase 1, degree = 5), SRSF5 (serine and arginine rich splicing factor 5, degree = 5), SRSF1 (serine and arginine rich splicing factor 1, degree = 5), HERC2 (HECT and RLD Domain Containing E3 Ubiquitin Protein Ligase 2, degree = 4), and TNPO1 (transportin 1). These five genes may play significant roles in the occurrence and development of SSBP.

### ROC Curve Analysis

**FIGURE 5:**
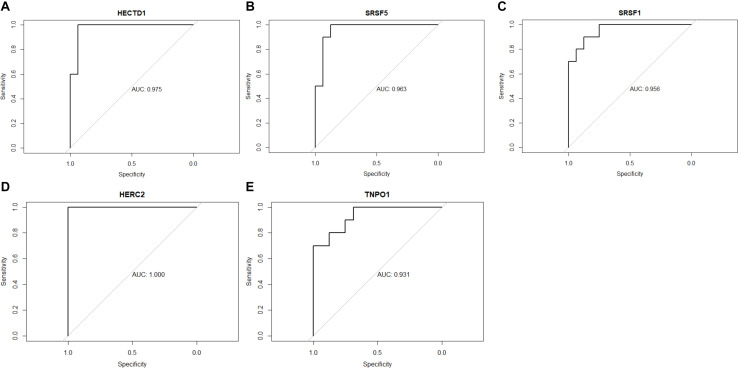
Receiver operating characteristic curves of the five-gene signature. **(A)** HECTD1. **(B)** SRSF5. **(C)** SRSF1. **(D)** HERC2. **(E)** TNPO1.

The ROC (receiver operating characteristic) AUCs (areas under the curve) of HECTD1, SRSF5, SRSF1, HERC2, and TNPO1 were calculated (Figure 5). The results showed that all three hub genes have high AUC of ROC: HECTD1 (AUC = 0.975), SRSF5 (AUC = 0.963), SRSF1 (AUC = 0.956), HERC2 (AUC = 1.000), and TNPO1 (AUC = 0.931), indicating that these five hub gene signatures have high diagnosis value and could serve as potential diagnosis biomarkers for SSBP.

### Identification of Differentially Expressed CircRNAs

**FIGURE 6:**
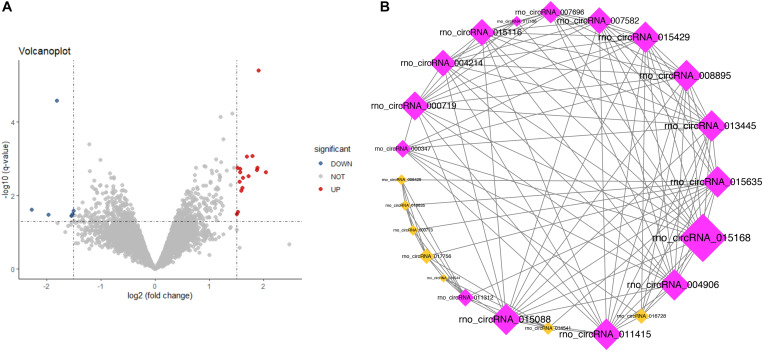
Volcano plot **(A)** and coexpression network **(B)** of the differentially expressed circRNAs (*P* < 0.05 and | log_2_FC| ≥ 1.5): red for upregulation and blue for downregulation.

CircRNAs with *P* <0.05 and | log_2_FC| ≥1.5 were considered as differentially expressed circRNAs (DECs). In the current study, based on the circRNAs’ expression profiles in GSE92669, we identified 23 DECs, including 16 upregulated and 7 downregulated DECs in the salt-sensitive group compared with the salt-resistant group (Figure 6A).

### Coexpression Network Analysis and Hub CircRNA Identification

In the present study, we constructed a circRNA coexpression network consisting of 2161 coexpression interactions among these 23 DECs based on the threshold of adjusted *P*-value (by BH method) <0.05 and | Pearson correlation| >0.9. The circRNA coexpression network is shown in Figure 6B. The top 5% circRNAs (one circRNA) in the coexpression network with the highest connectivity degree were identified as hub circRNAs: rno_circRNA_015168 (connectivity degree = 15). In the further analysis, we adopted a more straightforward naming approach based on the host genes of this hub circRNA—circAnks1b.

### Quantitative Real-Time Polymerase Chain Reaction (RT-qPCR) Validation

**FIGURE 7:**
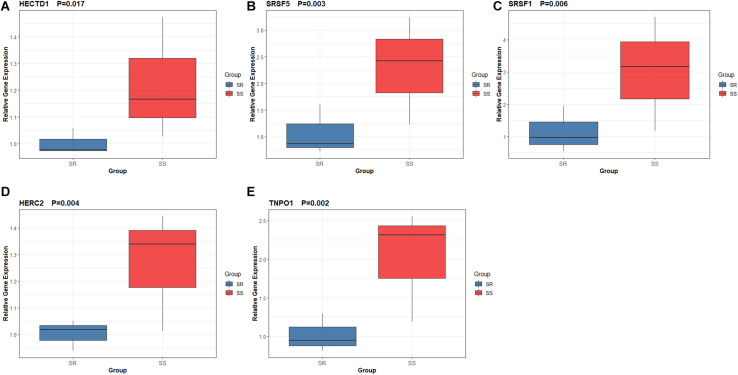
Expression level of five hub genes by quantitative real-time PCR. **(A)** HECTD1. **(B)** SRSF5. **(C)** SRSF1. **(D)** HERC2. **(E)** TNPO1.

In the present study, we performed quantitative RT-qPCR to detect the expression level of five hub genes with high connectivity degree in the PPI network and high AUC of ROC curves. Our results showed that the expression of HECTD1 (*P* = 0.017), SRSF5 (*P* = 0.003), SRSF1 (*P* = 0.006), HERC2 (*P* = 0.004), and TNPO1 (*P* = 0.002) were significantly upregulated in the renal tissue in salt-sensitive rats compared to salt-resistant rats, which indicated that HECTD1, SRSF5, SRSF1, HERC2, and TNPO1 can serve as potential biomarkers for SSBP (Figure 7).

## Discussion

SSBP is defined as the parallel changes of blood pressure in response to the change of salt intake, which is regarded as a significant risk factor for cardiovascular morbidity and mortality (Gu et al., 2018; Cuevas et al., 2020). However, the lack of an efficient biomarker and an unclear molecular mechanism of SSBP make it difficult to achieve early detection and intervention for high-risk individuals (Qi et al., 2018). CircRNAs and mRNAs are suggested to play important roles in various kinds of diseases and possess the ability to serve as diagnostic and prognostic biomarkers (Vilades et al., 2020; Zhang et al., 2020). Hence, we employed comprehensive bioinformatics approaches to select circRNAs and mRNA biomarkers for SSBP, which could provide novel insight into the molecular mechanism of SSBP.

In the current study, we identified a crucial module and 60 hub genes significantly associated with SSBP at the transcriptome level by WGCNA. Correlation analysis showed that the blue module is highly relevant to SSBP (Pearson correlation coefficient = 0.83), and most genes in this module have ideal GS and MM. To explore the functions of these 60 hub genes, GO functional enrichment analysis was carried out, and the results showed that these hub genes were significantly enriched in BPs such as artery development, cardiac development, and regulation of RNA splicing, as well as MF, including mRNA binding, microtubule binding, and tubulin binding. Then, we constructed a PPI network and identified several hub genes with high connectivity degrees in the PPI network that may serve as important regulators in SSBP. HECTD1 (HECT Domain E3 Ubiquitin Protein Ligase 1), HERC2 (HECT and RLD Domain Containing E3 Ubiquitin Protein Ligase 2), RNF14 (Ring Finger Protein 14), and UBR1 (Ubiquitin Protein Ligase E3 Component N-Recognin 1) all belong to the E3 ubiquitin-protein ligase family, which plays significant roles in ubiquitination protein modification by transferring ubiquitin from the E2 ubiquitin-conjugating enzyme to target substrates (Wang et al., 2017). Ubiquitination is a crucial post-translational regulation mechanism that modulates endogenous protein stability and PPIs (Yang et al., 2016; Cai et al., 2018). Some previous studies have shown that ubiquitination is involved in the process of sodium transport and the development of SSBP (Araki et al., 2009; Needham et al., 2011; Ronzaud et al., 2013). Our results may help to obtain a deeper understanding of the association between ubiquitination and sodium transport as well as SSBP. SRSF5 (serine- and arginine-rich splicing factor 5), SRSF1 (serine- and arginine-rich splicing factor 1), PNN (pinin, desmosome-associated protein), and PNISR (PNN-interacting serine- and arginine-rich protein) were considered to have marked effects on the regulation of the RNA AS process (Bush et al., 2017; Latorre and Harries, 2017). AS is a mechanism by which a single gene can generate multiple different transcripts and subsequently produce various proteins (Xu et al., 2018). A large number of studies on the association of RNA AS and hypertension have been reported, however, little attention has been focused on the correlation between AS and SSBP (Hu et al., 2017; Dlamini et al., 2019). Our results provided novel insight for further research on this aspect. Moreover, we found five hub genes with high diagnostic value by ROC curve analysis: HECTD1 (AUC = 0.975), SRSF5 (AUC = 0.963), SRSF1 (AUC = 0.956), HERC2 (AUC = 1.000), and TNPO1 (AUC = 0.931). Further validation experiment confirmed the differential expression of hub genes between salt-sensitive and salt-resistant rats, indicating that these five hub genes can serve as diagnostic biomarkers for SSBP.

In addition, one SSBP-associated hub circRNA (circANKS1B) was identified by combining coexpression network analysis and a DEC screening method. CircANKS1B has been demonstrated to be involved in several diseases, like breast cancer and colorectal cancer (Zeng et al., 2018; Li et al., 2019). In Zeng et al.’s (2018) research, they found that circANKS1B could induce epithelial-to-mesenchymal transition (EMT) via activating TGF-β1 signaling pathway in breast cancer. Our previous study demonstrated that salt intake could induce tubular EMT in Dahl/SS rat (Wang et al., 2014). In conclusion, circANKS1B might be a biomarker for SSBP. Although circular RNAs are attracting a good deal of attention, there has been little research in the field of circRNAs correlated with SSBP until now. Lu et al. (2020) found that CircNr1h4 is involved in the pathological process of salt-sensitive hypertension with renal injury by sponging miR-155-5p as a ceRNA. Additionally, Cheng and Joe (2017) compared the circRNA expression of the Dahl salt-sensitive rat, the Dahl salt-resistant rat, the spontaneously hypertensive rat, and the Wistar Kyoto rat by microarray analysis. Hence, our study identified a novel circRNA biomarker for SSBP and provided potential molecular targets for further research.

It should be noted that the present study did not verify the expression of the hub circRNA by molecular biology experiments. However, to increase the reliability of our results, we combined circRNA coexpression network analysis and DEG analysis. Notwithstanding this limitation, our study is the first to identify circRNA and mRNA biomarkers as well as a five-gene signature with high diagnostic value for SSBP based on comprehensive bioinformatics analysis. Hence, this study could narrow the scope and provide novel insight for further research focused on the molecular mechanism of SSBP.

## Materials and Methods

### Data Preprocessing

The gene expression profiles of GSE12424 and circRNA expression data of GSE92669 (Cuevas et al., 2020) were downloaded from the Gene Expression Omnibus database (GEO database)^1^. These gene expression profiles of GSE12424 were submitted by Andrew John McSweeny and consisted of 10 SS/jr rat kidney gene expression profiles and 8 congenic substrain S.R rat (D3Mco36-D3Mco46) kidney gene expression profiles as well as 8 congenic substrain S.R rat (D3Mco36-D3Got166) kidney gene expression profiles. S.R. rats (D3Mco36-D3Mco46) contain one blood pressure quantitative trait locus (QTL)-containing regions in the q-terminus of rat chromosome 3 (QTL2), while S.R. rats (D3Mco36-D3Got166) contain two blood pressure QTL-containing regions in the q-terminus of rat chromosome 3 (QTL1 and QTL2). These rats were all fed on a low-salt diet until 39–41 days of age and then half of the rats from each strain were randomly selected and changed to a high-salt diet. GSE92669 includes the circRNA expression data of three kidney samples of the Dahl salt-sensitive rat and three kidney samples of the Dahl salt-resistant rat. We then used the “impute” package in R software to impute the missing data by K-Nearest Neighbor (KNN) algorithm and the “limma” package to normalize the gene expression profiles. Meanwhile, the duplicated data were merged by selecting the median, and the data that could not be annotated were eliminated.

### Construction of a Weighted Gene Coexpression Network

The top 75% of genes with the highest MAD were selected for further analysis. The R package “WGCNA” was used to establish weighted gene coexpression networks (Langfelder and Horvath, 2008). First, we generated a similarity matrix according to the person correlation analysis between each pair of genes. Second, to achieve scale-free topology, we calculated an appropriate soft-thresholding power β using the “pickSoftThreshold” function in the “WGCNA” package. In this study, we set the threshold as scale-free index *R*^2^ >0.85. Third, the weighted adjacency matrix was derived.

### Identification of the Clinically Significant Modules and Hub Genes

We applied the “blockwiseModules” function in the “WGCNA” package to cluster genes into gene modules based on hierarchical clustering and a dynamic tree-cutting algorithm. The major parameters were as follows: maxBlockSize = 5000 and minModuleSize = 30. Furthermore, the MEs of each gene module were selected by a principal component analysis approach, and their expression levels were considered as the representatives of the expression of corresponding modules. Finally, we calculated Pearson’s correlation between each ME and clinical phenotypes. The modules with high correlation coefficients were used for further analysis, and the top 5% of genes in a module with the highest connectivity degree were identified as hub genes.

### Identification of DECs

The screening of DECs was performed by the “limma” package in R software. CircRNAs with *P* <0.05 and | log_2_FC| ≥1.5 were identified as DECs between the salt-sensitive group and the salt-resistant group.

### GO Functional Enrichment Analysis

In this study, we performed GO functional enrichment analysis and visualized the enriched BP, MF, and CC through the “clusterProfiler” package in R software. The thresholds were set as *P* <0.05 (Yu et al., 2012).

### PPI Network Analysis

A PPI network was constructed according to the Search Tool for the Retrieval of Interacting Genes database (STRING, version 11.0) with the threshold as interaction score >0.4. The visualization for the PPI network of these hub genes was achieved using Cytoscape (version 3.7.2).

### CircRNA Coexpression Network Analysis

The coexpression network analysis of DECs was conducted using the “psych” package in R software. We set the coexpression threshold as the adjusted *P*-value (by BH method) <0.05 and | Pearson correlation| > 0.9. The circRNA coexpression network was visualized by Cytoscape software. The top 5% circRNAs in the coexpression network with the highest connectivity degree were identified as hub circRNAs.

### ROC Curve Analysis

We performed ROC curve analysis based on “pROC” package in R software (Robin et al., 2011). The area under the curve (AUC) was calculated to evaluate the model performance.

### Rat Kidney Tissue Collection

Three Dahl Salt-Sensitive (Dahl/SS) rats and three SS-13BN rats at the age of 8 weeks were purchased from Charles River Laboratories International Inc. (United States). All rats were fed *ad libitum* at 25 ± 3°C with a 12-h light/12-h dark cycle in the specific-pathogen-free (SPF) animal room of Xi’an Jiaotong University. After being fed with a normal salt (0.3% NaCl) diet for 4 weeks, all rats were sacrificed and their kidneys were collected, stripped of fascia, flushed with saline, snap-frozen in liquid nitrogen, and then stored at −80°C until further experiment. The Ethics Committee of First Affiliated Hospital of Xi’an Jiaotong University granted ethical approval to this study (Ethical Approval Number: XJTU1AF2018G-062).

### Quantitative RT-qPCR Analysis

Total tissue RNA was extracted with the TRIzol reagent (Invitrogen) according to the manufacturer’s protocol. RevertAid First Strand cDNA Synthesis Kit (Thermo Fisher Scientific) was used to conduct reverse transcription. Then, qRT-PCR was performed on CFX Connect Real-Time PCR Detection System (BIO-RAD) with SYBR Green PCR Master Mix (Thermo Fisher Scientific). Finally, we analyzed the result via 2-ΔΔCt method and normalized the expression data using β-actin (as endogenous reference genes). The primer sequences are shown in Table 1.

**TABLE 1:** Primers used in quantitative real-time PCR experiments.

	Sequence
**β -actin**	
Forward	5-GGAGATTACTGCCCTGGCTCCTAGC-3
Reverse	5-GGCCGGACTCATCGTACTCCTGCTT-3
**HECTD1**	
Forward	5-TCCAAGCCCTGCTCACT-3
Reverse	5-CCAACCGAAAGCGTAAT-3
**SRSF5**	
Forward	5-TTGAGTTTGCCTCTTATGG-3
Reverse	5-GTGACCTGCTTCTTGACC-3
**SRSF1**	
Forward	5-CTGGTGTCGTGGAGTTTGT-3
Reverse	5-GGGAGAATAGCGTGGTGA-3
**HERC2**	
Forward	5-AAGTCTCCCAAGGATAAA-3
Reverse	5-GCACAGACGGAAGTAAA-3
**TNPO1**	
Forward	5-GGCTGGTGACGAGGAAG-3
Reverse	5-ACGGGAATCAACTTAGGC-3

## Conclusion

We identified 1 hub circRNA and 60 hub genes significantly associated with SSBP. Meanwhile, we put forward a five-gene signature with high diagnostic value by ROC curve analysis, which had been validated in the RT-qPCR experiments. In conclusion, our study found several potential biomarkers and provided a comprehensive understanding of SSBP.

## Data Availability Statement

The gene expression profiles of GSE12424 and circRNA expression data of GSE92669 were downloaded from the Gene Expression Omnibus database (GEO database; https://www.ncbi.nlm.nih.gov/geo/).

## Ethics Statement

The animal study was reviewed and approved by the Ethics Committee of the First Affiliated Hospital of Xi’an Jiaotong University.

## Author Contributions

J-JM and CChe: conception and design. CChu, YW, QM, K-KW, YYa, and Y-YL: collection and assembly of data. G-ZL, CChe, and W-LZ: analysis and interpretation of the data. CChe, G-ZL, and Y-YL: validation experiments. CChe, G-ZL, YYu, and J-WH: draft of the article. All authors contributed to the article and approved the submitted version.

## Conflict of Interest

The authors declare that the research was conducted in the absence of any commercial or financial relationships that could be construed as a potential conflict of interest.

## Footnotes

1 https://www.ncbi.nlm.nih.gov/geo/

## Supplementary Material

TABLE S1 Hub genes of salt sensitivity of blood pressure.
