# Supplementary material for: Identification of Candidate Biomarkers for Salt Sensitivity of Blood Pressure by Integrated Bioinformatics Analysis
Source: Front Genet. 2020 Sep 3;11:988. doi: 10.3389/fgene.2020.00988 (PMC7494969; doi:10.3389/fgene.2020.00988)
Supplement: TABLE S1 — Hub genes of salt sensitivity of blood pressure. [file Table_1.DOCX]

**Supplementary** **Table S1.** Hub genes of salt sensitivity of blood pressure

|  | hub_gene |  | hub_gene |  | hub_gene |
| --- | --- | --- | --- | --- | --- |
| 1 | Fat1 | 21 | Zbtb43 | 41 | Ythdf3 |
| 2 | Ltbp1 | 22 | Fyttd1 | 42 | Phf2 |
| 3 | Kdr | 23 | Nrtn | 43 | Lats2 |
| 4 | Lrp2 | 24 | Zc3h7a | 44 | Herc2 |
| 5 | Clasp2 | 25 | Reck | 45 | Exosc9 |
| 6 | Ets1 | 26 | Aff4 | 46 | Ccdc47 |
| 7 | Srsf5 | 27 | Tmem161b | 47 | Tnpo1 |
| 8 | Myh10 | 28 | Vps13b | 48 | Tcof1 |
| 9 | Ptp4a1 | 29 | Cd164 | 49 | Il6st |
| 10 | Mylk | 30 | Dync1i2 | 50 | Wapal |
| 11 | Dpysl2 | 31 | Spag9 | 51 | Ankrd17 |
| 12 | Ahnak | 32 | Man2a1 | 52 | Col5a2 |
| 13 | Reep3 | 33 | Ccser2 | 53 | Pnisr |
| 14 | Rev3l | 34 | Slfn5 | 54 | Son |
| 15 | Myef2 | 35 | Tns3 | 55 | Rhoq |
| 16 | Hectd1 | 36 | Supt20 | 56 | Sec63 |
| 17 | Nbr1 | 37 | Rnf14 | 57 | Srsf1 |
| 18 | RGD1308143 | 38 | Adck4 | 58 | Pnn |
| 19 | LOC678893 | 39 | Ubr1 | 59 | Yme1l1 |
| 20 | Cnot6 | 40 | Sbno1 | 60 | Zmiz1 |
